# Abstracts/Sommaires/Zusammenfassungen

**DOI:** 10.1155/S1463924680000387

**Published:** 1980


### Abstracts Sommaires Zusammenfassungen

### Page 57

**Relative costing of analytical systems**
H.G.J. Worth

A method of costing analysis is proposed which enables the costs of carrying out analyses for a given workload using different instruments to be compared. The procedure is kept as simple as possible by only considering those costs which may vary from one analyser to another, in so doing a number of assumptions are made which have to be justified. Each part of the costing analysis is described in detail and then applied to ten instruments which are capable of carrying out similar analyses. Examples of analytical systems, using combinations of these instruments, are compared to illustrate the usefulness of the costing procedure. The conclusions are that a relatively simple costing analysis has been devised whereby instrument running costs may be compared for a given workload. However, its full application can only be realised through a computer program which then enables many instruments in different analytical systems to be compared and applied to different workloads.

**Frais relatifs des systèmes analytiques**

Une méthode d'analyse de frais est proposée, que permet la comparaison des frais d'analyses pour un travail bien défini, en employant différents instruments. La procédure est tenue aussi simple qui possible, en considérant uniquement les frais qui puissent varier d'un analysateur à un autre, en agissant ainsi un certain nombre de suppositions sont faites qui doivent être justifiées. Chaque partie de l'analyse de frais est décrite en détail et en suite appliquée à dix instruments, qui sont capables d'exécuter des analyses similaires. Exemples de systèmes analytiques, utilisant des combinations de ces instruments, sont comparés afin d'illustrer l'utilité de la procédure de frais. En conclusion, on peut dire qu'une analyse de frais relativement simple a été conçue, qui permet la comparaison des frais courants d'un instrument pour un travail défini. Néanmoins, son application complète ne peut être réalisée que par ordinateur, ce qui permettra la comparaison de beaucoup d'instruments travaillant selon différents systèmes analytiques, exécutant des travaux différents.

**Relative Kosten eines analytischen Systems**

Eine Methode zur Analyse der Kosten wird vorgeschlagen, die erlaubt die Kosten für die Durchführung von Analysen bei vorgegebener Arbeitslast für verschiedene Instrumente miteinander zu vergleichen. Das Vorgehen is so einfach wie möglich gestaltet, indem nur diejenigen Kosten betrachtet werden, die vom einen zum anderen Analysator andern. Dabei müssen eine Reihe von Annahmen gemacht werden, die zu rechfertigen sind. Jedser Teil der Kostenanalyse ist im Detail beschrieben und wird auf zehn Instrumente angewendet, welche ähnliche Analysen durchführen können. Beispiele von analytischen Systemen bestehend aus Kombinationen dieser Instrumente, werden miteinander verglichen um die Nützlichkeit der Kostenanalyse zu demonstrieren. Die Schlussfolgerungen sind, dass eine relativ einfache Kostenanalyse entstanden ist, bei der bei vorgegebener Arbeitslast die laufenden Kosten für die Instrumente verglichen werden können. Den vollen Nutzen wird man jedoch erst mit Hilfe eines Computer-Programms realisieren, welches dann den Vergleich vieler Instrumente in verschiedenen analytischen Systemen und unter Verschiedener Arbeitslast erlaubt.

### Page 134

**Design considerations for an automated hydride evolution system based on continuous flow principles**
A.L. Dennis and D.G. Porter

Because of their potential toxicity, it is necessary to be able to accurately measure environmental concentrations of certain elements, such as arsensic. It has been found that adequate sensitivity can be achieved using an automated hydride evolution procedure, and the development of an automated system using this method is described.

**Considérations pour un projet de système automatique pour dégagement d'hydrides basé sur les principes de flux continu**

A cause de leur toxicité potentielle, il est nécessaire de mesurer avec beaucoup de précision la concentration dans l'environnement de certains éléments, p.e. l'arsenic. On a constaté qu'une sensibilité adéquate peut être atteinte en employant une procédure automatique pour dégagement d'hydrides, et l'emploi d'un système automatique selon cette méthode est décrit.

**Konstruktionsbetrachtungen für ein automatisiertes Hydrid-Evaluationssystem auf der Basis des kontinuierlichen Flusses**

Wegen ihrer potentiellen Toxizität ist es notwendig, dass die Konzentrationen in der Umwelt gewisser Elemente wie Arsen gemessen werden können. Es wurde festgestellt, dass eine genügende Empfindlichkeit mit einem automatisierten Hydrid-Evaluationsverfahren erhalten wird. Die Entwicklung eines automatisierten Systems basierend auf dieser Methode wird beschrieben.

### Page 139

**An automatic method for the determination of bromide in water**
R.E.D. Moxon and E.J. Dixon

An indirect method for the determination of bromide in natural waters has been automated. The method is based on the catalytic effect of bromide on the oxidation of iodine to iodate by permanganate, the excess iodine being extracted into carbon tetrachloride and measured colorimetrically. Problems associated with the automation of a system involving solvent extraction were examined and the method was applied to a range of United Kingdom drinking waters. The method has a precision in the order of five percent, a detection limit of 4 $\mu$g per litre, a recovery of added bromide ranging from 95 to 108 percent and an output of 20 samples per hour. The effect of possible interfering substances has been investigated.

**Une méthode automatique pour la détermination de bromure dans l'eau**

Une méthode indirecte pour la détermination de bromure dans de l'eau naturelle a été automatisée. La méthode est basée sur l'effet catalique de bromure sur l'oxidation d'iodure en iodate du permanganate, l'iodure en excès est extrait dans du tétrachlorure de carbone et mesurée de façon colorimétrique. Les problèmes associés à l'automation d'un système utilisant l'extraction par solvents ont été examinés et la méthode a été appliquée à une série d'eaux potables du Royaume Uni. La méthode a une précision d'environ cinq pourcent, une limite de détection de 4 $\mu$g par litre, une récupération du bromure ajouté de 95 à 108 pourcent et une capacité de 20 échantillons par heure. L'effet de substances pouvant interférer a été recherché.

**Eine automatische Methode zur Bestimmung von Brom in Wasser**

Eine indirekte Methode für die Bestimmung von Brom in natürlichen Gewässern wurde automatisiert. Die Methode basiert auf der katalytischen Wirkung von Brom auf die Oxydation von Iod zu Iodat durch Permanganat, Extraktion des überschüssigen Iods mit Tetrachlorkohlenstoff und kolorimetrische Bestimmung. Die Probleme, die im Zusammenhang mit der Automatisierung eines Systems mit Extraktion durch Lösungsmittel entstehen, wurden untersucht, und die Methode wurde auf eine Reihe von britischen Trinkwässer angewandt. Die Methode ergibt eine Präzision von ungefähr 5%, eine Empfindlichkeitsgrenze von 4 $\mu$g/l, eine Erfassung des enthaltenen Broms zwischen 95 und 108 Prozent und einen Durchsatz von 20 Proben pro Stunde. Ferner wurde die Wirkung möglicherweise störender Substanzen untersucht.

### Page 143

**An evaluation of the Beckman Astra 8 analyser**

P.H. Lloyd, H. Bicknell and P.M.G. Broughton

An evaluation has been made of the Beckman ASTRA-8, a seven-channel, automatic discrete analyser, which will analyse serum for urea, sodium, potassium chloride, total carbon dioxide, glucose and creatinine. Apart from creatinine, all analyses are made by electrochemical methods.

**Une évaluation de l'analysateur Beckman Astra 8**

Une évaluation a été faite du Beckman ASTRA-8, analysateur automatique discret à sept canaux qui analyse le sérum pour l'urea, le soude, la potasse, le chlorure, le dioxyde de carbone total, le glucose et la créatinine. Mise à part la créatinine, toutes les analyses sont faites à l'aide de méthodes électrochimiques.

**Eine Evaluation des Beckman Astra 8 Analysators**

Eine Evaluation des 7-Kanals automatischen Proben-Analysator Beckman ASTRA-8 fur die Analyse von Harnstoff, Na, K, Cl, Gesamt-$CO_2$, Glukose und Kreatinin wurde durchgeführt. Abgesehen von Kreatinin erfolgten alle Analysen elektrochemisch.

### Page 149

**Particle counting immunoassay (PACIA) V— its application to the determination of human placental lactogen**

A.E. Leek, F. de Steenwinkel, C.L. Cambiaso and P.L. Masson

Particle Counting Immunoassay (PACIA) has been applied to the determination of human placental lactogen using a completely automated system, with a total throughput time of 31 min. The threshold sensitivity in the best conditions was 0.1 µg/l and the practical range in serum from 0.5 mg/l to 20 mg/l; the correlation coefficient with radioimmunoassay was 0.99; the inter-assay coefficient of variation was less than 6.5%.

**Immunoassay par comptage de particules (PACIA) V— son application pour la détermination de lactogène humain placentaire**

Immunoassay par comptage de particules (PACIA) a été appliqué pour la détermination de lactogène humain placentaire, en employant un système complètement automatique, avec un temps de passage total de 31 min. La limite de détection dans les meilleures conditions était de 0.1 µg/l, et la rangée pratique de sérum de 0.5 mg/l à 20 mg/l; le coéfficient de corrélation avec radioimmunoassay était 0.99; le coéfficient de variation entre différents essais était de moins de 6.5%.

**Particle counting immunoassay (PACIA) V— Anwendung auf die Bestimmung von menschlichem Plazenta-Lactogen**

Particle Counting Immunoassay (PACIA) wurde für die Bestimmung von menschlichen Plazenta-Lactogen benützt unter Verwendung eines vollständig automatisierten Systems mit einer Gesamtdurchlaufzeit von 31 Minuten. Die Schwellenempfindlichkeit betrug unter besten Vehältnissen 0.1 µg/l und der praktisch benützbare Serumsbereich 0.5 mg/l bis 20 mg/l; der Korrelationskoeffizient mit Radioimmunoassay ergab 0.99; der Variationskoeffizient zwischen den Versuchen war weniger als 6.5%.

### Page 153

**Evaluation of the C parallel analyser**

R. Puukka and M. Puukka

A new analyser, C Parallel Analyser, was evaluated for the routine clinical measurement of seven laboratory tests. The factors evaluated for ALP, AST, alpha-amylase, albumin, cholesterol, creatinine and triglycerides were as follows: 1. rate of analysis of patient sample, 2. overall precision (within-day and day-to-day), 3. analytical linearity, 4. correlation of values with those obtained by methods used routinely in our laboratory, 5. error detection messages, 6. reliability.

Experience indicates that the C Parallel Analyser can analyse about 40-60 patient samples for one component in 15 minutes, incubation periods excluded. It is also easy to operate. The within-day and day-to-day precisions (coefficient of variance) obtained were good, generally less than 3.0% and 5.0%, respectively. The ranges of linearity were wide enough for every analysis and the correlation studies indicated a good agreement with normal routine laboratory methods. Furthermore, the free choice of dispensing order and volumes, incubation times and measuring mode enable a great flexibility of the analyser in chemical methods.

**Evaluation de l'analyseur parallèle C**

Un nouvel analyseur, l'analyseur parallèle C, a été évalué pour mesurages cliniques de routine de sept tests de laboratoiré. Les facteurs évalués pour ALP, AST, alpha-amylase, albumine, cholestérine, créatinine et triglycérides ont été les suivants: 1. temps d'analyse d'un échantillon de patient, 2. précision totale (du même jour et de jour en jour), 3. linéarité analytique, 4. corrélation des valeurs avec celles obtenues par des méthodes de routine dans notre laboratoire, 5. communication des erreurs détectées, 6. fiabilité.

L'expérience prouve que l'analyseur parallel C peut analyser environ 40-60 échantillons de patient pour un composant en 15 minutes, periodes d'incubation exclues. Il est très facile à employer. Les précisions du même jour et de jour en jour (coefficient de variation) obtenues étaient bonnes, en général moins de 3.0% et 5.0%, respectivement. Les plages de linéarité étaient assez larges pour chaque analyse et les études de corrélation indiquent une bonne entente avec des méthodes de laboratoire de routine. En plus, le libre choix d'ordres de dosage et volumes, temps d'incubation et mode de mesurage permettent une grande flexibility de l'analyseur en méthodes chimiques.

**Evaluation des C Parallel-Analysators**

Ein neuer Analysator, der C Parallel-Analysator, wurde für die klinische Routinebestimmung von sieben Laboruntersuchungen evaluiert. Die untersuchten Faktoren für die Bestimmung von ALP, AST. alpha-Amylase, Albumin, Cholesterol, Kreatinin und Triglyzerid waren: 1. Analysengeschwindigkeit der Patientenprobe, 2. Gesamtpräzision (täglish und von Tag-zu-Tag), 3. analytische Linearität, 4. Korrelation mit Werten, die in unserem Laboratorium mit den bestehenden Routinemethoden erhalten werden, 5. Fehlermeldungen, 6. Zuverlässigkeit.

Die Erfahrungen ergeben, dass der C Parallel-Analysator ungefähr 40-60 Patientenproben auf eine Komponente in 15 Minuten analysieren kann, wobei die Inkubationszeit nicht berücksichtigt ist. Der Analysator ist einfach zu bedienen. Die beobachtete tägliche und Tag-zu-Tag Präzision (Variationskoeffizienten) ist gut, im allgemeinen kleiner als 3.0% bzw. 5.0%. Die Linearitätsbereiche sind für alle Analysen gross genug und Korrelationsstudien ergaben eine gute Uebereinstimmung mit den bestehenden Routine-Laboratoriumsmethoden. Die freie Wahl der Dosier-Reihenfolge und -Volumina, der Inkubations-Zeiten und der Messart ergeben eine grosse Flexibilität für die chemischen Methoden des Analysators.

### Page 159

**An improved automated colorimetric analysis of fructose in fermentation media.**

R.B. Roy and A. Buccafuri

An automated method is described for the rapid analysis of glucose-isomerising activity in fermentation broths. It retains the accuracy of previous methods (such as the 'Dische reaction') while using fewer modules and milder reaction conditions. Twenty samples per hour may be analysed, with fructose in the range of 0.01-0.1 mg/ml.

**Une analyse colorimetrique automatique améliorée de fructose en milieu de fermentation**

Une méthode automatique est décrite pour analyse rapide d'activité d'isomérisation de glucose en fermentation de bouillon. Elle a la précision des méthods antérieures (p.e. la "réaction Dische") parce qu'elle utilise moins de modules et des conditions de réaction plus légères. Vingt échantillons par heure peuvent être analysés, avec un fructose de 0.01-0.1 mg/ml.

**Eine verbesserte automatisierte kalorimetrische Analyse auf Fruktose in Fermentationsmischungen**

Eine automatisierte Methode zue raschen Bestimmung der Glukose-Isomerisierungsaktivität in Fermentationsmischungen wird beschrieben. Diese erreicht die Genauigkeit bisheriger Methoden (wie z.B. die "Dische-Reaktion") aber unter Benützung einer kleineren Zahl von Modulen und unter milderen Reaktionsbedingungen. Zwanzig Proben mit Fruktose im Bereich von 0.01-0.1 mg/ml können pro Stunde analysiert werden.

